# Deep Learning in Urological Images Using Convolutional Neural Networks: An Artificial Intelligence Study

**DOI:** 10.5152/tud.2022.22030

**Published:** 2022-07-01

**Authors:** Ahmet Serel, Sefa Alperen Ozturk, Sedat Soyupek, Huseyin Bulut Serel

**Affiliations:** 1 Department of Urology, Suleyman Demirel University School of Medicine, Isparta, Turkey; 2 Software Quality Assurance Engineer, Getir, Ankara, Turkey

**Keywords:** Deep learning, machine learning, artificial intelligence, hydronephrosis, vesicoureteral reflux

## Abstract

**Objective:**

Using artificial intelligence and a deep learning algorithm can differentiate vesicoureteral reflux and hydronephrosis reliably.

**Material and Methods:**

An online dataset of vesicoureteral reflux and hydronephrosis images were abstracted. We developed image analysis and deep learning workflow. The images were trained to distinguish between vesicoureteral reflux and hydronephrosis. The discriminative capability was quantified using receiver-operating characteristic curve analysis. We used Scikit learn to interpret the model.

**Results:**

Thirty-nine of the hydronephrosis and 42 of the vesicoureteral reflux images were abstracted from an online dataset. First, we randomly divided the images into training and validation. In this example, we put 68 cases into training and 13 into validation. We did inference on 2 cases and in return their predictions were predicted: [[0.00006]] hydronephrosis, predicted: [[0.99874]] vesicoureteral reflux on 2 test cases.

**Conclusion:**

This study showed a high-level overview of building a deep neural network for urological image classification. It is concluded that using artificial intelligence with deep learning methods can be applied to differentiate all urological images.

**Main Points**

- Artificial intelligence is gaining popularity in whole fields of science.
- In order to catch up with the era, physicians must have sufficient knowledge on this subject.
- Our model successfully differentiates the hydronephrosis versus vesicoureteral reflux images
- With the help of artificial intelligence, generating successful clinical adjuncts improves urologic clinical practice.

## Introduction

Machine learning (ML) algorithms have been applied to medical imaging, and their use is increasing day by day in the field of medical sciences. Deep learning (DL), in particular, has shown to be more efficient in image assessment and processing. Deep learning algorithms may help and simplify their use in urological imaging. This article introduces how to create a convolutional neural network (CNN) algorithm for urological image analysis. Deep learning, a sub-branch of ML, includes multi-layered neural networks. Convolutional neural network has been used extensively in image classification and data processing.^1^ It has been applied to image categorization firstly by Krizhevsky et al.^2^ They won a competition with a deep CNN called AlexNet in the 2012 ImageNet Large Scale Visual Recognition Challenge (ILSVRC), consisting of 1.2 million everyday color images.^3^ In another CNN model, Lakhani et al^4^ demonstrated that they made an accurate algorithm to distinguish chest vs. abdominal radiographs in medical imaging. 

However, its use in urological imaging has been developing, and the studies concerned with DL and CNN are limited. Thus, this study is designed to present DL algorithms for purposes of urological image classification. We anticipate that this study will serve as a primary starting point for further research into DL in the field of urological imaging. Our work is based on Python 3.6 (Python 3.6, Python Software Foundation, Wilmington, DE, USA) coding program, designed to provide a helpful approach to using libraries and frameworks.^5^

## Material and Methods

Artificial intelligence (AI) ML is applied to the dataset for distinguishing 2 groups from each other (e.g., vesicoureteral reflux (VUR) and hydronephrosis(HN)). Our dataset contains 81 images (39 images representing HN and 42 images for VUR).

The data were acquired from OpenI, the National Institutes of Health’s online open archive of medical images from published PubMed Central papers (https://openi.nlm.nih.gov). Those images were previously diagnosed as VUR or HN with VCUG and intravenous pyelography. The images were converted to the Portable Network Graphics format for using the ML system if necessary. Since all the data used were obtained from an open-access archive and all analyses were performed on these data, bringing an informed consent form and ethics committee approval was not necessary.

Numerous functional libraries ease ML research and development, such as Caffe, MXNet, Tensorflow, Theano, Torch, and PyTorch.^6^ Tensorflow framework (Tensorflow 1.4, Google LLC, Mountain View, Calif, USA) and the Keras library (Keras v 2.12, https://keras.io/) were chosen for our study.^7^

Initially, the images must be randomly split into training and validation set groups. The training group consisted of 68 cases, and the validation group consisted of 13 cases. These references provide access to the principles of our study model and boosted the methodologies.^8,9^ Tensorflow, Keras, and Jupyter Notebook (http://jupyter.org/) were used to set up our data for this study; it is an open-source web tool that allows you to create and share documents containing text and live code.^10^ Firstly, Keras library prerequisites were loaded into the Jupyter notebook. Subsequently, the information about the images was set. The number of epochs (number of training data processed) and the batch size (number of images processed simultaneously) were determined. The model was constructed, and then the pre-trained Inception V3 network was created.^11^ Since Keras has 2 possible categories (HN or VUR radiograph), the binary cross-entropy loss was utilized as the model performance criterion with a probability between 0 and 1. On the other hand, for classification works, binary cross-entropy was used.

Many existing gradient descent optimization methods are available to reduce the workload of a given objective procedure.^12^ The Adam optimizer was preferred in this study with generally employed settings.^13^ Transfer learning has been documented in ML as applying a procedure designed for 1 task to a different challenge.^14^ As a result, radiographs can be categorized with ML models that have been trained to identify photographs such as ordinary colored pictures.

All images have comparable properties like margins and lines, and they can help achieve transfer learning. Massive datasets (in the billions) are required to train deep neural networks successfully. Therefore, using large dataset networks that have been trained before is better for small sample-sized studies to obtain higher performance.^14–16^ It might be challenging to describe a vast dataset to train from scratch, particularly in healthcare imaging classification algorithms.

Two primary transfer learning tactics are freezing and training on the last layers with a low learning rate. The proper method is often found by trial and error learning. Therefore, the modeling should be done with different options. In this study, a few additional layers with random initialization were inserted into the final Inception V3 model, which was pre-trained in ImageNet before. Thus, the new medical data enabled these layers to learn from them. A fine-tuned model was used with a low learning rate (0.0001). It did not distort the weights, which are already relatively well optimized.

Feature engineering of images includes preprocessing and defining augmentation options, such as modifications and other image interpretations, which might assist avoid selection bias and memorization of the training dataset. Kanishka et al^17^ have previously demonstrated that they can improve the precision and understanding of CNNs. Keras contains data generator tools to modify the images in Python programming. Thus it is required to import to the network just before. After that, the training directory comprising the files, image size, and batch size is given to the generator. Thus, the final codes for the model running are put into the generator.

We used AI for the differentiation of HN versus VUR images. The AI compared images, not numbers, so the classical statistical analysis could not perform for this study.

The required analyses were performed with the Sci-Kit library in Python 3.6 (Python 3.6, Python Software Foundation) coding program.

## Results

The model begins to train after the code has been run. The accuracy of the validation and training was 95% and 100%, respectively, after only 6 epochs.

**Figure 1.**
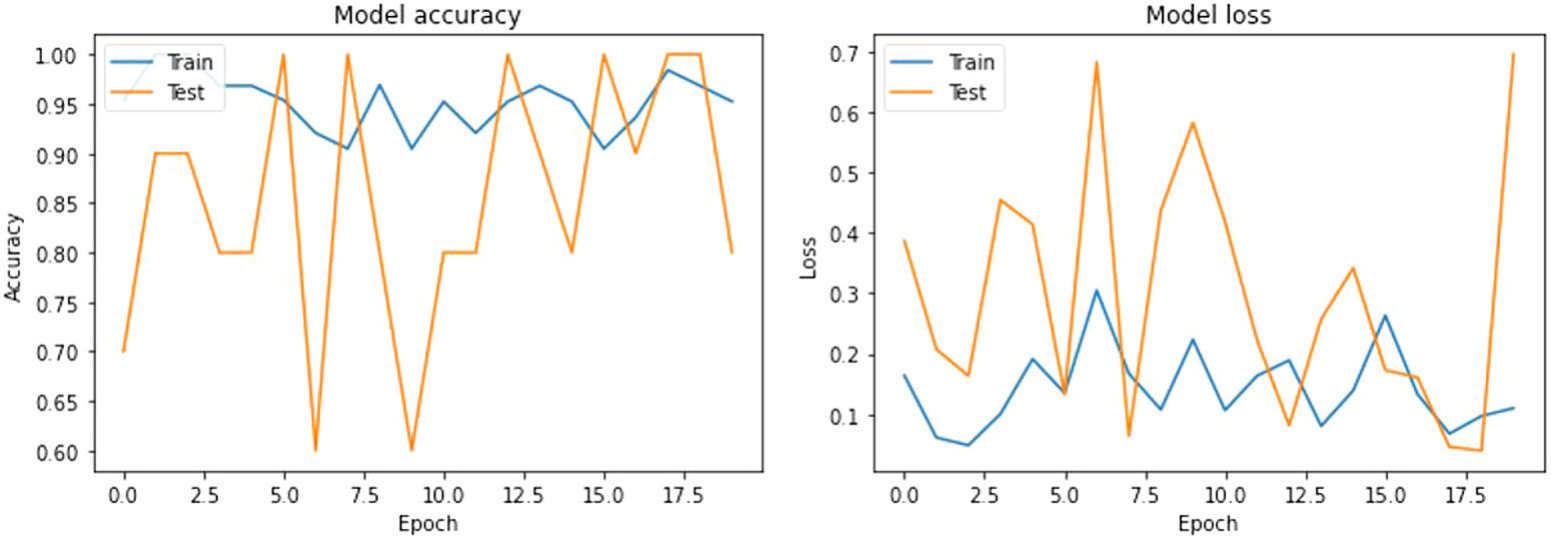
Sample Python code to plot training data. Accuracy increases and loss decreases over time for the training and validation.

Contrary to our study, validation accuracy is measured lower than the training accuracy. The cause of this is the lower number of cases (n = 13) in the validation group. The decrease in the loss of validation and training means that the model is “learning.” Figure 1 shows the Matplotlib graphic, a plotting library in Python software, and produces the figures in various formats. The values concerning loss and accuracy are recorded in arrays with Matplotlib.

**Figure 2.**
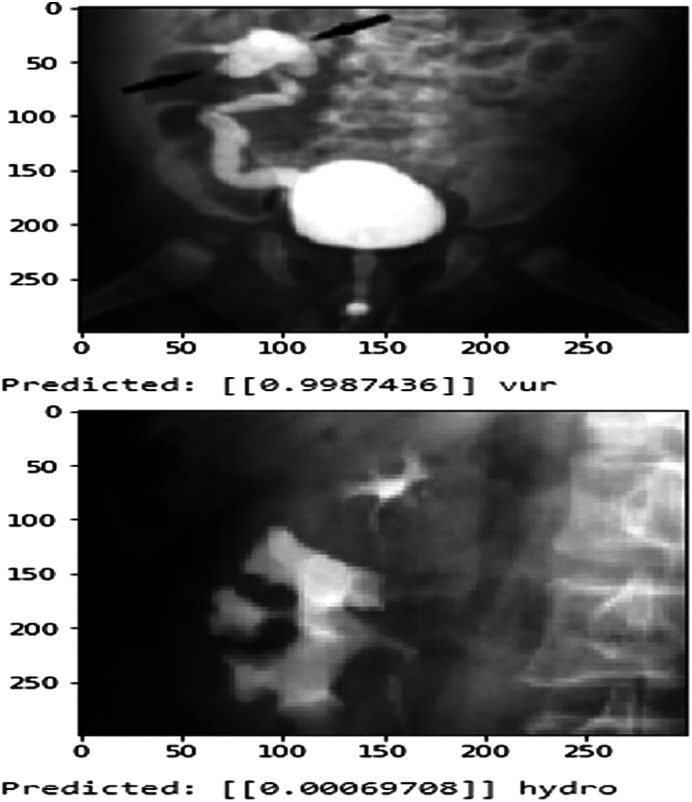
Inference on 2 test cases. The numbers within the brackets represent the probability of hydronephrosis versus vesicoureteral reflux (range 0-1). A score close to 0 indicates high confidence of hydronephrosis, and a score close to 1 indicates high confidence of vesicoureteral reflux.

To evaluate the performance of the trained model, held-out test cases are required in addition for a better sense of generalization, in addition to training and validation of images. Keras library could use the data generator on a batch of test cases, use a for-loop on an entire directory of cases, or evaluate 1 case at a time. In the presented work, we used 2 test cases and predicted their probabilities. Figure 2 shows the predicted [[0.00006]] HN and predicted [[0.99874]] VUR on the test cases. The numbers within the brackets represent the probability of HN versus VUR radiograph (range 0-1). A score close to 0 indicates high confidence of a HN radiograph, and a score close to 1 indicates high confidence of a VUR radiograph.

Sci-Kit library in Python or a separate statistical program could generate a receiver-operating characteristic curve with the outputs from the model.

## Discussion

Our study demonstrated whether AI could accurately recognize VUR and HN. Overall, our model was able to categorize images correctly. This DL model showed accurate discrimination between VUR and HN with external validation in a simple-to-use web application. With the help of a small amount of code and training cases, an accurate differentiating algorithm could recognize and classify HN versus VUR radiographs that shows us the power of DL. The availability of frameworks and high-level libraries may help DL to be more accessible in urological imaging. It is concluded that this study may provide a foundation for starting with DL algorithms in urological imaging. The principal finding of this study is that DL can be applied to standardize and automate various urological images easily. Further research is needed to employ CNN studies to evaluate HN or VUR images. Also, in another study, we are currently working on, we believe that AI will achieve to classify the grade of VUR perfectly. In the future, we will completely exclude the interobserver bias for images with the help of AI, that is, more cost-effectivity and reduced radiation exposure for the patients by preventing unnecessary imaging. In addition, both the time of surgery can be predicted more accurately, and the success of treatment can be evaluated more objectively.

The major limitation is that we studied with the data that were only publicly sourced images, and it was also limited by training a model based on subjective data without proven reliability. We believe that by adding the extensive multi-institutional urological image database and the other quantitative measures here, the more robust artificial intelligence and DL model may be possible to predict directly by correlating with clinically relevant outcomes of urological images.

In conclusion, our DL model demonstrated that it could generate successful clinical adjuncts to improve clinical practice. Urological imaging using AI is possible, even in non-standardized datasets of images. Therefore, DL algorithms can be applied to all urological images.
